# Adherence to Treatment Guidelines and Associated Survival in Older Patients with Prostate Cancer: A Prospective Multicentre Cohort Study

**DOI:** 10.3390/cancers13184694

**Published:** 2021-09-18

**Authors:** Adolfo González Serrano, Claudia Martínez Tapia, Alexandre de la Taille, Pierre Mongiat-Artus, Jacques Irani, Axel Bex, Elena Paillaud, Etienne Audureau, Thomas Barnay, Marie Laurent, Florence Canouï-Poitrine

**Affiliations:** 1 Univ Paris Est Creteil, INSERM, IMRB, F-94010 Creteil, France; claudia.tapia-ext@aphp.fr (C.M.T.); alexandre.de-la-taille@aphp.fr (A.d.l.T.); elena.paillaud@aphp.fr (E.P.); etienne.audureau@aphp.fr (E.A.); marie.laurent@aphp.fr (M.L.); florence.canoui-poitrine@aphp.fr (F.C.-P.); 2 Department of Urology, AP-HP, Hôpital Henri Mondor, F-94010 Creteil, France; 3 Université de Paris, INSERM UMR_S1165, F-75010 Paris, France; pierre.mongiat-artus@aphp.fr; 4 Department of Urology, AP-HP, Hôpital Saint Louis, F-75010 Paris, France; 5 Faculty of Medicine, Université Paris Saclay, F-94270 Le Kremlin-Bicêtre, France; jacques.irani@aphp.fr; 6 Department of Urology, AP-HP, Hôpital Bicêtre, F-94270 Le Kremlin-Bicêtre, France; 7 Division of Surgery and Interventional Science, University College London, London NW3 2QG, UK; a.bex@ucl.ac.uk; 8 Specialist Centre for Kidney Cancer, Royal Free London NHS Foundation Trust, London NW3 2QG, UK; 9 Department of Geriatrics, Paris Cancer Institute CARPEM, AP-HP, Hôpital Européen Georges Pompidou, F-75006 Paris, France; 10 Faculty of Health, Univeristé de Paris, F-75006 Paris, France; 11 Department of Public Health, AP-HP, Hôpital Henri Mondor, F-94010 Creteil, France; 12 ERUDITE Research Unit, Univ Paris Est Creteil, F-94010 Créteil, France; barnay@u-pec.fr; 13 Department of Internal Medicine and Geriatrics, AP-HP, Hôpital Henri Mondor, F-94010 Creteil, France

**Keywords:** aged, age factors, clinical decision-making, geriatrics, guideline adherence, practice guidelines, prostatic neoplasms

## Abstract

**Simple Summary:**

According to international guidelines, the treatment of prostate cancer in men aged 70 or over should consider the patient’s health status. Although these guidelines were published more than ten years ago, the level of their implementation in routine clinical practice has not been evaluated. Here, we studied the degree of implementation of these guidelines and the association between their implementation and patients’ survival. We found that the selected treatment was not guideline-compliant in almost half the patients, that non-metastatic disease was a factor related to non-compliance with the guidelines and that non-compliance with the guidelines was associated with worse survival.

**Abstract:**

The guidelines on prostate cancer treatment in older men recommend evaluating the patient’s underlying health status before treatment selection. We aimed to evaluate the frequency of a guideline–discordant treatment (GDT), identify factors associated with GDT, and assess the relationship between GDT and overall survival. We studied patients with prostate cancer aged 70 or older included in the ELCAPA cohort between 2010 and 2019. Multivariable logistic regression assessed GDT-associated factors. The restricted mean survival time (RMST) assessed the 24- and 36-month OS using stabilized inverse probability of treatment weighting of propensity scores. We included 356 patients (median age: 81 years), and 164 (46%) received a GDT (95% confidence interval (CI) = (41–51%)). Patients with metastases were less likely to receive a GDT (adjusted odds ratio (95% CI) = 0.34 (0.17–0.69); *p* = 0.003). After weighting, the RMST at 24 months was shorter in the GDT group (13.9 months, vs. 17 months for compliant treatments; difference (95% CI): −3.1 months (−5.3, −1.0); *p* = 0.004). RMST at 36 months was 18.5 months, vs. 21.8 months (difference: −3.3 months (−6.7, 0.0); *p* = 0.053). GDT is common in older patients with prostate cancer and especially those with non-metastatic disease. GDT was associated with worse survival, independently of health status and tumour characteristics.

## 1. Introduction

Prostate cancer primarily affects older men; worldwide, it is the most commonly diagnosed cancer and the second leading cause of cancer death among men aged 70 and over [1]. The incidence of prostate cancer in this age group is expected to rise by 35% between 2018 and 2030 [2].

Cancer treatment in older patients is complicated by age-related health conditions, such as comorbidities, functional impairments, and frailty [3,4]. These factors influence treatment decisions, increase treatment toxicity, and diminish overall survival (OS) [5,6]. Within the same age group, health status and life expectancy vary from one patient to another [7]. Hence, the choice of treatment must balance the corresponding risks and benefits, based on factors other than the patient’s age [7]. Thus, a geriatric assessment (GA, a multidimensional assessment of the risk of frailty) is now recommended as a guide to the most appropriate treatment [8]. Based on the GA results, Balducci and Extermann first defined three groups of older patients with cancer: group 1 were functionally independent patients with no serious comorbidities, group 2 were dependent in one instrumental activity of daily living and had one or two potential comorbidities, and group 3 were frail patients (dependent in one or more activities of daily living, with three or more comorbid conditions or one or more geriatric syndromes) [9].

In 2010, the International Society of Geriatric Oncology (SIOG) published specific guidelines on treating prostate cancer in men aged 70 years and over [10]. These guidelines defined four groups according to the GA’s results (fit, vulnerable, frail, and terminal) and recommended that treatment decisions should be stratified by group. First, fit patients can receive standard treatments. Vulnerable patients can receive standard treatments but may benefit from additional geriatric care. Treatment should be adjusted in frail patients, and additional geriatric care is required. Lastly, terminal patients should receive supportive care. The SIOG guidelines were updated in 2014 [11], 2017 [12], and 2019 [13] and have been endorsed by learned societies such as the European Association of Urology (EAU) and the French Association of Urology (AFU).

Although these guidelines have been widely disseminated and there is evidence of a correlation between guideline-discordant treatment (GDT) and poorer outcomes in older patients with other types of cancer [14,15], the level of implementation in routine clinical practice has not been evaluated. Hence, the objectives of the present real-life study were to (i) estimate the frequency of GDT in patients with prostate cancer aged 70 or over, (ii) identify factors associated with GDT, and (iii) evaluate the relationship between GDT and OS at 24 and 36 months.

## 2. Materials and Methods

### 2.1. Design and Patients

ELCAPA is an ongoing, prospective, multicentre cohort study of patients aged 70 or over with a confirmed diagnosis of cancer and who have been referred to a geriatric oncology clinic for a GA before the choice of cancer treatment. After the GA, a multidisciplinary meeting including geriatricians, medical oncologists, urologists, and radiation oncologists is held for treatment selection. The participating geriatric oncology clinics are in 19 hospitals in the greater Paris area of France. The GA used in the ELCAPA study has been described previously [5]. The present study was performed in accordance with the Declaration of Helsinki. All patients provided their informed consent before inclusion. The study protocol was approved by an independent ethics committee (CPP Ile-de-France I, Paris, France; reference: 2019 mai-MS121). ELCAPA is registered at ClinicalTrials.gov (NCT02884375).

In April 2009, the AFU published its first guidelines on treating prostate cancer in older men [16]. We considered that it would have taken a further 12 months before the guidelines were considered during GAs. Hence, for this study (ELCAPA-26), we analysed patients with prostate cancer included in the ELCAPA study between April 2010 and November 2019. According to a previous study, the estimated prevalence of GDT among older patients with prostate cancer was 50% [17]. To estimate this prevalence with an accuracy of 0.05 and a two-sided type 1 error of 0.05, we calculated that 384 patients would have to be included in our analysis.

### 2.2. Outcomes

The primary outcome was GDT, and the secondary outcomes were 24- and 36-month OS. A GDT was defined as a selected treatment that was not guideline-compliant regarding the patient’s health status. We first defined three binary variables: the presence or absence of GDT according to the SIOG [10,11,12], EAU [18,19,20,21,22,23,24], and AFU guidelines [16,25,26,27] over the period from 2009 to 2018. For instance, considering that it would have taken a further 12 months before the guidelines were considered for treatment selection, for a patient included in 2015, the health status and the corresponding treatment was assessed using the AFU guidelines of 2013, the SIOG guidelines of 2014 and the EAU guidelines of 2014. Next, we defined a fourth binary variable as GDT if the selected treatment was not in compliance to at least one of the three sets of guidelines. We accounted for differences in the year of publication, the instruments used for health status assessment and classification, and the recommended treatment (Table S1).

GDT was considered to be a lower-intensity treatment if the patient was treated less intensively than recommended by the guidelines. Conversely, GDT was considered to be a higher-intensity treatment if the patient was treated more intensively than recommended by the guidelines (Figure S2).

OS was defined as the time interval between the GA and all-cause death recorded during the first 24 and 36 months of follow-up. The choice of these time horizons relied on the fact that previous studies showed that the mean age of older patients consulting in geriatric oncology clinics is 79.6 years, and their mean number of comorbidities is 4.2 [5]. Conversely, the median age at death for patients with prostate cancer in France is 83 years [27], and the life expectancy of prostate cancer patients aged 80 years, with four comorbidities and localized disease, is six years, whereas for metastatic disease is 1.5 years, respectively [28].

### 2.3. Follow-Up

Follow-up started at the date of GA and ended on the date of death, the date of data extraction (27 December 2019), or (for censored patients) the date of the last follow-up, whichever came first.

### 2.4. Covariates

We considered (i) tumour-related variables, including the TNM stage (non-metastatic or metastatic), Gleason score, the serum prostate-specific antigen (PSA), type of treatment (surgery, radiotherapy ± androgen deprivation therapy (ADT), chemotherapy and supportive care), (ii) GA variables, including the 8-item G8 frailty screening tool, the Activities of Daily Living (ADL) score, the Instrumental Activities of Daily Living (IADL) score, the Eastern Cooperative Oncology Group performance status (ECOG PS), the Cumulative Illness Rating Scale for Geriatrics (CIRS-G) score, the risk of depression and cognitive impairment (defined according to the geriatrician’s clinical judgment but guided by the results of the mini Geriatric Depression Scale and Mini-Mental State Examination, respectively), weight loss during the previous three months, smoking status, marital status, and living alone status (a patient living alone in an individual housing unit), and (iii) vital status (based on medical records or data from the public records office) at 24 and 36 months of follow-up. The GA variables were assessed by an experienced geriatrician, using a standardized case report form.

### 2.5. Statistical Analysis

#### 2.5.1. Evaluation of GDT

The study population’s demographic characteristics were described with summary statistics. The degree of agreement between the three sets of guidelines was evaluated using Fleiss’ k statistic (95% CI).

To identify factors associated with GDT, we performed a bivariate analysis by using a chi-squared test for categorical variables and a t test for continuous variables. The data for the serum PSA level was not normally distributed and so were log-transformed before the bivariate analysis. Variables with *p* < 0.2 in the bivariate analysis were fed into the multivariable analysis. The adjusted odds ratios (aORs) (95% CI) for GDT were estimated using multivariable logistic regression.

#### 2.5.2. Survival Analysis

To evaluate factors associated with 24- and 36-month OS, we applied the log-rank test to categorical variables and a Cox proportional hazards regression to continuous variables. Crude hazard ratios (HRs) (95% CI) were estimated using a bivariate Cox proportional hazards regression. The Schoenfeld residuals slope test and plots were used to assess the proportional hazards assumption. The intergroup difference in the mean expected time to death was calculated as the restricted mean survival time (RMST), based on the areas under the survival curves for the GDT group and the guideline-concordant treatment (GCT) group for a given time horizon. Unlike the HR, the RMST does not require the proportional hazards assumption [29].

Treatment allocation frequently depends on patient’s characteristics in observational studies. In consequence, the differences in baseline characteristics between treatment groups can influence treatment results. Therefore, the estimation of treatment effects must account for these differences. Methods based on propensity scores are intended to account for these differences [30]. To minimize confounding and selection biases when evaluating the association between GDT and survival, we applied stabilized inverse probability of treatment weighting (SIPTW) to propensity scores.

#### 2.5.3. Estimation of the Propensity Score and Stabilized Weights

First, we imputed missing data by using multivariate imputation with chained equations under the missing-at-random assumption, which generated 10 imputed data sets. Second, for each data set, we fitted a logistic regression model to estimate the probability of receiving GDT (the propensity score) and thus obtained pooled estimates according to Rubin’s rules [31]. This model included all the variables associated with GDT and 24- and 36-month OS. Third, we calculated stabilized weights in order to reduce the influence of propensity score outliers [32]. To this end, we used logistic regression to determine the probability of receiving GDT without considering covariates (pGDT). SIPTW was defined as (pGDT/propensity score) if patients received GDT or (1 − pGDT/(1 − propensity score)) if not. The covariate balance between groups after weighting was checked against the standard mean difference: a value <0.1 indicated good covariate balance [32]. Violations of the positivity clause and misspecification of the propensity score model were checked using the mean of the stabilized weights: a value far from 1 indicated violation of the positivity clause or model misspecification [32]. Fourth, we used a SIPTW-adjusted Kaplan-Meier technique to assess intergroup differences in 24- and 36-month OS; the resulting weighted HRs and RMSTs were quoted with their 95% CIs.

### 2.6. Sensitivity Analysis

We performed the analyses for each set of guidelines. Furthermore, we included the centre and the year of inclusion in the propensity score model, to account for differences in these variables. We also analysed the data regarding treatment intensity, clinical stage and geriatric risk profile (G8 score ≤ 14). A specific pseudopopulation was built (using SIPTW) for each sensitivity analysis. Lastly, we calculated the E-value to assess the effect of potential unmeasured confounding in the assessment of GDT. The E-value estimates the strength required by unmeasured confounders to explain away the observed effects [33].

All statistical tests were two sided. The threshold for statistical significance was set to *p* < 0.05. All statistical analyses were performed using Stata software (version 14.2, StataCorp, College Station, TX, USA).

## 3. Results

### 3.1. Study Population

**Figure 1:**
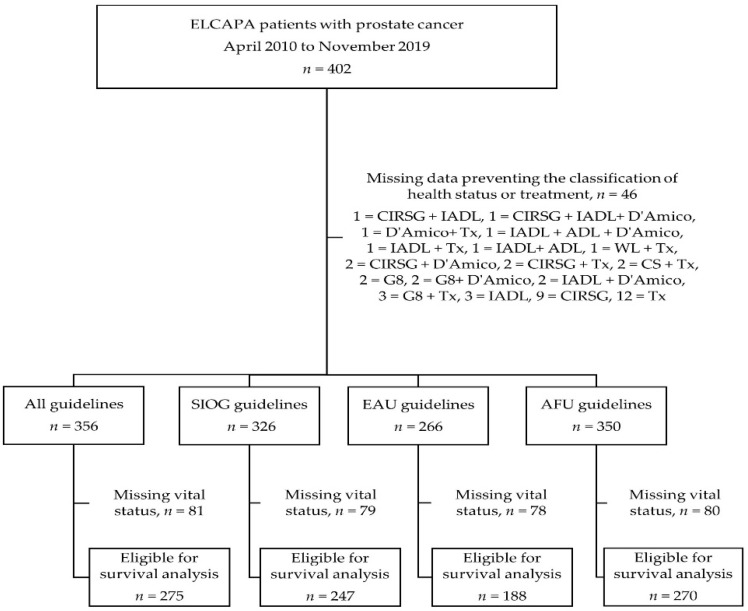
Flow diagram of participants and available data, depending on the set of guidelines. ADL: Activities of Daily Living score, AFU: French Association of Urology, CIRS-G: Cumulative Illness Rating Scale for Geriatrics, CS: clinical stage, D’Amico: D’Amico risk group classification system, EAU: European Association of Urology, IADL: Instrumental Activities of Daily Living score, SIOG: International Society of Geriatric Oncology, Tx: treatment, WL: weight loss.

Between 8 April 2010 and 27 November 2019, 402 patients with prostate cancer were included in the ELCAPA study (Figure 1). The median (interquartile range (IQR)) age was 81 (77–85), 252 patients (63%) had metastatic disease, and the median serum PSA level was 30 ng/mL (10.3–122). Three hundred and sixty-two patients (90%) presented at least one abnormal GA variable. Fifty-four patients (13%) were considered to be fit, 81 (20%) vulnerable, 230 (57%) frail, and 30 (8%) terminally ill. Other characteristics of the study population are summarized in Table S2.

### 3.2. Evaluation of GDT

Data on treatments and health status were available for 356 of the 402 patients (89%). One hundred and sixty-four of these 356 patients (46%; 95% CI = (41–51%)) received GDT. There was a substantial degree of agreement between the three sets of guidelines for GDT classification (κ (95% CI) = 0.71; (0.66–0.77)). The treatments included radical prostatectomy in 14 patients (4%), radiotherapy in 63 (18%), ADT (as monotherapy or as an adjuvant) in 198 (56%), and chemotherapy in 95 patients (27%). Supportive care was chosen for 42 patients (12%) (Figure S1). Among the patients with GDT, 54 (33%) received lower-intensity treatment and 110 (67%) received higher-intensity treatment. In the latter group, 56 patients (51%) did not have metastatic disease and 54 had metastatic disease (49%) (Figure S2). The proportion of patients with GDT fell slightly over the study period but increased transiently after updated SIOG guidelines were published. (Figure S3).

Mobility, age, the risk of depression, a cognitive impairment, the Gleason score, the serum PSA level, and the clinical stage were considered in our multivariable analysis. The results of the bivariate analyses are summarized in Table S3. After adjustment, metastatic disease was still associated with a lower probability of GDT (aOR (95% CI) = 0.34 (0.17–0.69); *p* = 0.003) (Table 1).

### 3.3. Survival

Follow-up data were available for 275 patients. The median (IQR) length of follow-up for survivors was 44 months (27–70). The overall probability (95% CI) of 24- and 36-month OS was 0.43 (37–49) and 0.37 (0.31–0.43), respectively. The GCT and GDT groups did not differ significantly regarding the unadjusted probability of 24- (0.39 (0.31–0.48) vs. 0.47 (0.38–0.55); logrank *p* = 0.8) and 36-month OS (0.33 (0.25–0.42) vs. 0.41 (0.33–0.50); logrank *p* = 0.8). In bivariate analyses, factors associated with poorer 24- and 36-month OS were G8 score ≤ 14, older age, restricted mobility, ≥1 abnormal ADL item, ≥1 abnormal IADL item, weight loss, a higher Gleason score, a higher serum PSA level, ECOG-PS, ≥1 CIRS-G grade 3 comorbidities or ≥1 CIRS-G grade 4 comorbidities, the risk of depression, cognitive impairment, and clinical stage (Table S4).

**Figure 2:**
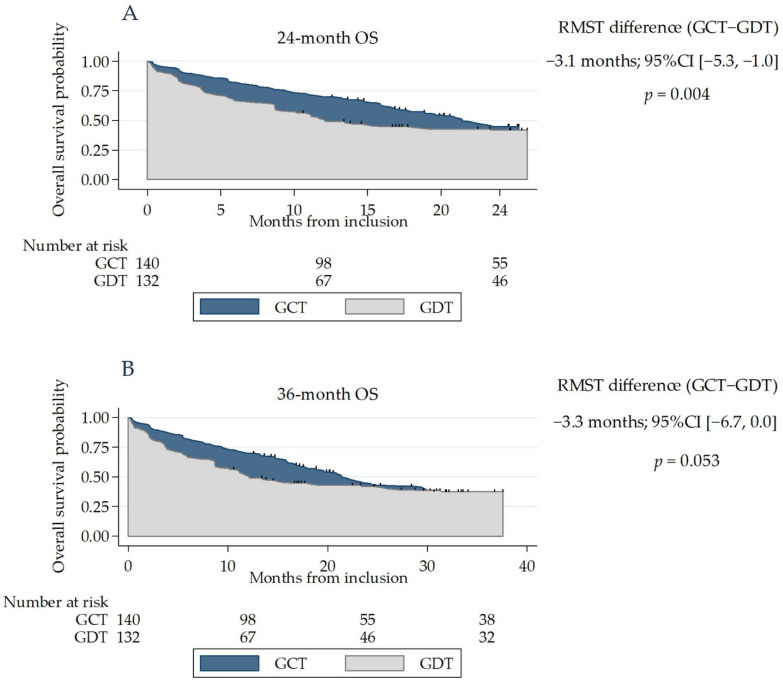
Kaplan–Meier curves for overall survival in the GDT and GCT groups. The area in blue represents the difference in the restricted mean survival time (RMST). (**A**) The weighted population, using SIPTW of propensity scores for 24-month OS. (**B**) The weighted population, using SIPTW of propensity scores for 36-month OS. GCT: guideline-concordant treatment, GDT: guideline–discordant treatment.

Weighting achieved a good covariate balance between the groups (Table 2). The risk of death did not differ significantly between groups at 24 (weighted HR (95% CI) = 1.31 (0.92–1.87); *p* = 0.1) and 36 months (weighted HR (95% CI) = 1.22 (0.87–1.71); *p* = 0.2). However, the proportional hazards assumption was not satisfied (*p* ≤ 0.01 for both of Schoenfeld residuals). The 24-month expected time to death was significantly shorter in the GDT group than in the GCT group (13.9 vs. 17 months, respectively; difference (95% CI): −3.1 months (−5.3, −1.0); *p* = 0.004). RMST at 36 months was 18.5 months, vs. 21.8 months (difference: −3.3 months (−6.7, 0.0); *p* = 0.053). (Figure 2).

### 3.4. Sensitivity Analyses

**Figure 3:**
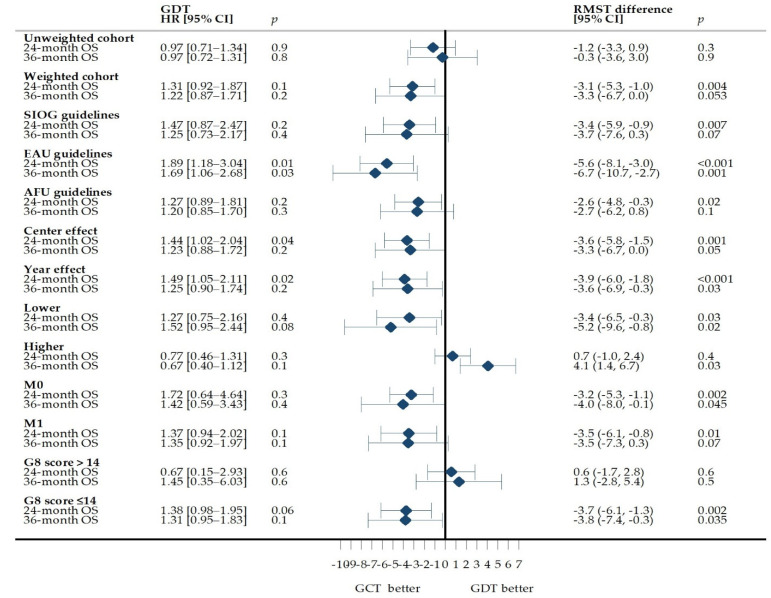
Association of GDT with 24- and 36-month overall survival and intergroup differences in OS before and after SIPTW. The difference in RMST is expressed in months. A negative RMST difference corresponds to a shorter survival time in the GDT group than in the GCT group. A specific pseudopopulation was generated using SIPTW for all cohorts other than the unweighted cohort. None of the HR estimations satisfied the proportional hazards assumption. AFU: French Association of Urology, EAU: European Association of Urology, GCT: guideline–concordant treatment, GDT: guideline–discordant treatment, Higher: higher-intensity treatment, HR: hazard ratio, Lower: lower-intensity treatment, M0: non-metastatic disease, M1: metastatic disease, SIOG: International Society of Geriatric Oncology, SIPTW: standardized inverse probability of treatment weights.

Similar results were observed when the data for each set of guidelines were analysed separately. The centres, the year of inclusion and the clinical stage did not change the association between GDT and survival. GDT in the lower-intensity treatment group and patients with a geriatric risk profile was associated with a significantly shorter 24- and 36-month expected time to death (Figure 3).

The E-value for the association between clinical stage and GDT was 2.82, with a lower limit of the CI of 1.70, which means that an unmeasured confounder requires an association with GDT and clinical stage by an OR of 2.82-fold each to explain away the observed association.

## 4. Discussion

In our cohort of 356 older men with prostate cancer, 164 (46%) received GDT and GDT was more likely in patients with non-metastatic disease. After follow-up, the mean survival time was shorter in the GDT group.

In the literature, the reported prevalence of GDT among prostate cancer patients aged 70 or over (31 to 37%) was lower than the value observed in the present study [34,35]. However, these population-based studies included younger patients (median age: 73), patients with non-metastatic disease only and defined GDT according to the National Comprehensive Cancer Network (NCCN) guidelines only. The latter guidelines do not consider the patient’s health status in the treatment selection, and the studies by Chen et al. [34] and Hamilton et al. [35] did not consider the patient’s health status in their definitions of GDT.

Our observation of a higher probability of GCT in patients with metastatic disease (aOR for GDT in metastatic disease (95% CI) = 0.34 (0.17–0.69); *p* = 0.003) contrasts with Ellis et al.’s [36] report of a lower probability of GCT in patients with metastatic disease (OR (95% CI) = 0.09 (0.03–0.31); *p* ≤ 0.001). However, the mean age in Ellis et al.’s cohort was 61 (vs. 81 in our study), and only 22 (3%) of their patients had metastatic disease (vs. 63% in our study). This difference may be explained by the fact that the SIOG, EAU, and AFU guidelines considered ADT to be a standard treatment in older men with metastatic, hormone-sensitive prostate cancer. Our results are also consistent with reports of greater guideline compliance in patients receiving ADT [34].

Several studies of prostate cancer have shown that GDT is more likely in older patients than in younger patients [34,35,36]. Our study included only patients aged 70 or over, and older age was not associated with GDT. Moreover, most of the previously cited studies defined GDT as a non-curative treatment for patients with localized disease. Furthermore, all but one of the studies used the NCCN guidelines to define discordance/concordance. The latter guidelines do not consider the patient’s health status in the treatment selection, and these studies did not take account of the patient’s health status in their definitions of GDT.

In our analyses, several geriatric variables were not associated with a GDT. A possible explanation for this absence of association is that awareness of geriatric issues may lead to physician and patient concerns about potential treatment consequences. A higher vigilance in delivering treatments frequently leads to geriatric interventions and adapted treatments in older patients with cancer [5,35]. However, an adapted treatment does not necessarily mean a guideline–concordant treatment. Proof of this is the 46% frequency of GDT observed in our study. Moreover, evaluating the level of awareness and endorsement of guidelines among physicians participating in multidisciplinary meetings for treatment selection would be necessary to support this hypothesis. This assessment was out of the scope of the present study and merits further evaluation.

Consistent with other studies, we found that higher-intensity treatment was common in the GDT group [37]. Higher-intensity treatment may result from (i) a misperceived, overly optimistic prognosis based on the GA results, (ii) the physician’s desire to provide treatment, (iii) the patients’ desire to undergo treatment, or (iv) unawareness of the GA results [38].

OS was worse in the GDT group than in the GCT group. Several studies have shown that older patients are more likely to receive less intensive care than is recommended [14,35]. However, the literature data concerning the relationship between GDT and OS are contradictory. In older patients, GDT (and especially less intensive care than recommended) has been linked to worse survival in certain studies [14,15], but not others [39]. Moreover, the data on overly intensive care are also contradictory [37]. The results of our sensitivity analyses suggested that worse OS applies to patients receiving less intensive care than recommended and to patients with a geriatric risk profile. Worse survival in these situations may result from treatment toxicity, treatment reduction or discontinuation [40].

Our study had limitations. First, certain treatments may have been misclassified about concordance or discordance with the guidelines. However, any such misclassification would have affected both GCT and GDT and would have been independent of the patient’s vital status. Thus, misclassification can be considered as a non-differential factor resulting in bias towards the null hypothesis [41]. The use of different guidelines for defining GDT might also have led to misclassification. However, the kappa statistic evidenced the substantial degree of agreement between the three sets of guidelines. Second, referral bias was possible because the ELCAPA patients were recruited by centres with expertise in geriatric oncology and this could have influenced our results. Despite this, our results are the best to be expected, as our patients come from centres with expertise in geriatric oncology. Outside these centres, results might be worse. Third, unmeasured confounding was possible because of unmeasured variables or variables not included in our multivariable regression model. However, the estimated E-value showed that an unmeasured variable would have to be associated at a OR of 2.82 or greater with clinical stage and GDT to explain away our findings. The probability of such a confounding variable is remote because the ORs for all the included risk factors in the bivariate analysis were lower than the calculated E-value. Lastly, data on the patient’s preferences potentially a determinant in treatment selection were not collected.

Our study also had strengths. First, we accounted for confounding and selection biases by applying SIPTW to propensity scores. Second, we evaluated three different sets of guidelines simultaneously; this may enable our findings to be more easily extrapolated to contexts in which these guidelines are employed.

Lastly, we accounted for changes in the definition of GDT and health status over time and thus gained an extensive overview of guideline use.

Evidence-based guidelines are supposed to improve the quality of medical care by supporting decision-making [42]. However, we observed a considerable level of guideline non-compliance, and the latter was associated with worse survival. The level of evidence underpinning SIOG and other consensus-based guidelines is low, which might hinder compliance [42,43]. Understanding physicians’ and patients’ judgments when making treatment decisions (e.g., in mixed methods studies) may provide useful information for optimizing guideline compliance.

Guidelines on treating older men with prostate cancer must be implemented more effectively. This issue is important because prostate cancer experts still believe that is not necessary to consider the patient’s health status when selecting a treatment for patients aged 70 or older [44,45].

## 5. Conclusions

Non-compliance with treatment guidelines (relative to the underlying health status) is common in older patients with prostate cancer in general and in those with non-metastatic disease in particular. GDT was associated with worse survival, independently of the patient’s health status and tumour characteristics.

## Figures and Tables

**Table 1:** Multivariable analysis of factors associated with GDT.

Variable	Category	Adjusted OR (95% CI)	
		(*n* = 190)	*p*
Mobility (G8 score item)	Goes out	1	
	Able to get out of bed/chair but does not go out	1.79 (0.51–6.25)	0.36
	Bed- or chair-bound	0.91 (0.37–2.26)	0.84
Age	<80	1	
	80–85	0.87 (0.35–2.15)	0.76
	>86	0.58 (0.28–1.18)	0.13
Risk of Depression	No	1	
	Yes	0.55 (0.28–1.1)	0.09
Cognitive Impairment	No	1	
	Yes	1.20 (0.56–2.53)	0.64
Gleason Score	≤6	1	
	7	0.73 (0.26–2.03)	0.55
	8 à 10	0.85 (0.32–2.25)	0.74
Serum PSA Level	Log PSA	0.96 (0.79–1.16)	0.64
Clinical Stage	Non-metastatic	1	
	Metastatic	0.34 (0.17–0.69)	0.003

Multivariable analysis using a logistic regression model that included the factors listed in the table: mobility, risk of depression, cognitive impairment, Gleason score, serum PSA level and clinical stage. CI: confidence interval, GDT: guideline–discordant treatment, OR: odds ratio, PSA: prostate-specific antigen.

**Table 2:** Baseline characteristics of the patients included in survival analyses before and after SIPTW.

Characteristic	Original Cohort			Weighted Cohort *		
	GCT (%)	GDT (%)	SMD	GCT (%)	GDT (%)	SMD
*n* = 275	*n* = 143	*n* = 132				
G8 score						
≤14	113 (80)	100 (77)	−0.04	78	78	−0.008
Activities of Daily Living score						
≥1 abnormal item	15 (10)	18 (14)	0.14	11	12	0.02
Instrumental Activities of Daily Living score						
≥1 abnormal item	55 (40)	43 (35)	0.2	40	39	−0.03
Mobility (G8 score item)						
Goes out	90 (66)	93 (74)	−0.04	70	69	−0.02
Able to get out of bed/chair but does not go out	31 (23)	17 (14)		19	19	
Bed- or chair-bound	15 (11)	15 (12)		11	12	
Age						
<80	57 (40)	76 (54)	−0.2	47	50	−0.07
81–90	71 (50)	57 (42)		46	43	
>90	15 (10)	5 (4)		7	7	
Cumulative Illness Rating Scale for Geriatrics grade 3 or 4 comorbidities						
At least one	93 (68)	92 (71)	0.15	70	70	−0.001
Eastern Cooperative Oncology Group performance status						
0–1	69 (49)	78 (59)	0.09	55	53	0.03
2	37 (26)	25 (19)		22	23	
3–4	36 (25)	29 (22)		23	24	
Risk of depression						
Yes	55 (41)	40 (31)	−0.3	38	37	−0.02
Cognitive impairment						
Yes	52 (37)	29 (23)	−0.12	32	31	−0.02
Weight loss						
<5%	88 (62)	87 (66)	−0.08	65	67	0.04
5–10%	29 (20)	18 (14)		20	12	
>10%	25 (18)	26 (20)		15	21	
Gleason score						
≤6	9 (10)	16 (16)	−0.2	11	9	0.06
7	31 (34)	42 (42)		35	36	
8 to 10	51 (56)	41 (41)		54	55	
Serum PSA level (median (IQR))						
Log PSA	3.7 (2.9–4.9)	3.1 (2.2–4.8)	−0.2	3.7 (2.8–4.8)	3.1 (2.2–4.6)	0.01
Clinical stage						
Metastatic	105 (73)	62 (48)	−0.3	63	62	−0.03

* A specific pseudopopulation (weighted cohort) was generated using SIPTW for each time horizon (24 and 36 months of follow-up). The distribution of baseline characteristics and SMD between groups were the same at 24 and 36 months of follow-up. Percentages only are presented for the weighted cohort. SMD < 0.1 indicates a good covariate balance between groups. The propensity score model used for SIPTW included all variables associated with GDT and 36-month OS (G8 score < 14, age, mobility, ≥1 abnormal ADL item, ≥1 abnormal IADL item, weight loss, the Gleason score, serum PSA level, ECOG-PS, ≥1 CIRS-G grade 3 comorbidities or ≥1 CIRS-G grade 4 comorbidities, depression, cognitive impairment, and clinical stage). GCT: guideline–concordant treatment, GDT: guideline–discordant treatment, PSA: prostate-specific antigen, SIPTW: stabilized inversed probability of treatment weighting, SMD: standardized mean difference.

## Data Availability

Restrictions apply to the availability of these data. Data was obtained from the ELCAPA Study Group and are available from the corresponding author with the permission of the ELCAPA Study Group investigators.

## Supplementary Materials

The following are available online at https://www.mdpi.com/article/10.3390/cancers13184694/s1, Figure S1: Treatments in the GDT and GCT groups, stratified by clinical stage and health status, Figure S2: Treatments in the GDT group, stratified by treatment intensity, clinical stage, and health status, Figure S3: Change over time (2010–2019) in the proportion of GDT in the ELCAPA cohort, Table S1: Variables used in the guidelines for health status definition, and the classifying algorithm, Table S2: Demographic characteristics of participants, Table S3: Bivariate analysis for receiving guideline–discordant treatment, according to at least one of the three sets of guidelines, Table S4: Bivariate analysis of predictors of overall survival at 24 and 36 months.
