# Supplementary material for: Adherence to Treatment Guidelines and Associated Survival in Older Patients with Prostate Cancer: A Prospective Multicentre Cohort Study
Source: Cancers (Basel). 2021 Sep 18;13(18):4694. doi: 10.3390/cancers13184694 (PMC8468518; doi:10.3390/cancers13184694)
Supplement: Supplementary file 1 [file cancers-13-04694-s001.zip › Figure S1.pdf]

Figure S1. Treatments in the GDT and GCT groups, stratified by clinical stage and health status ‡.

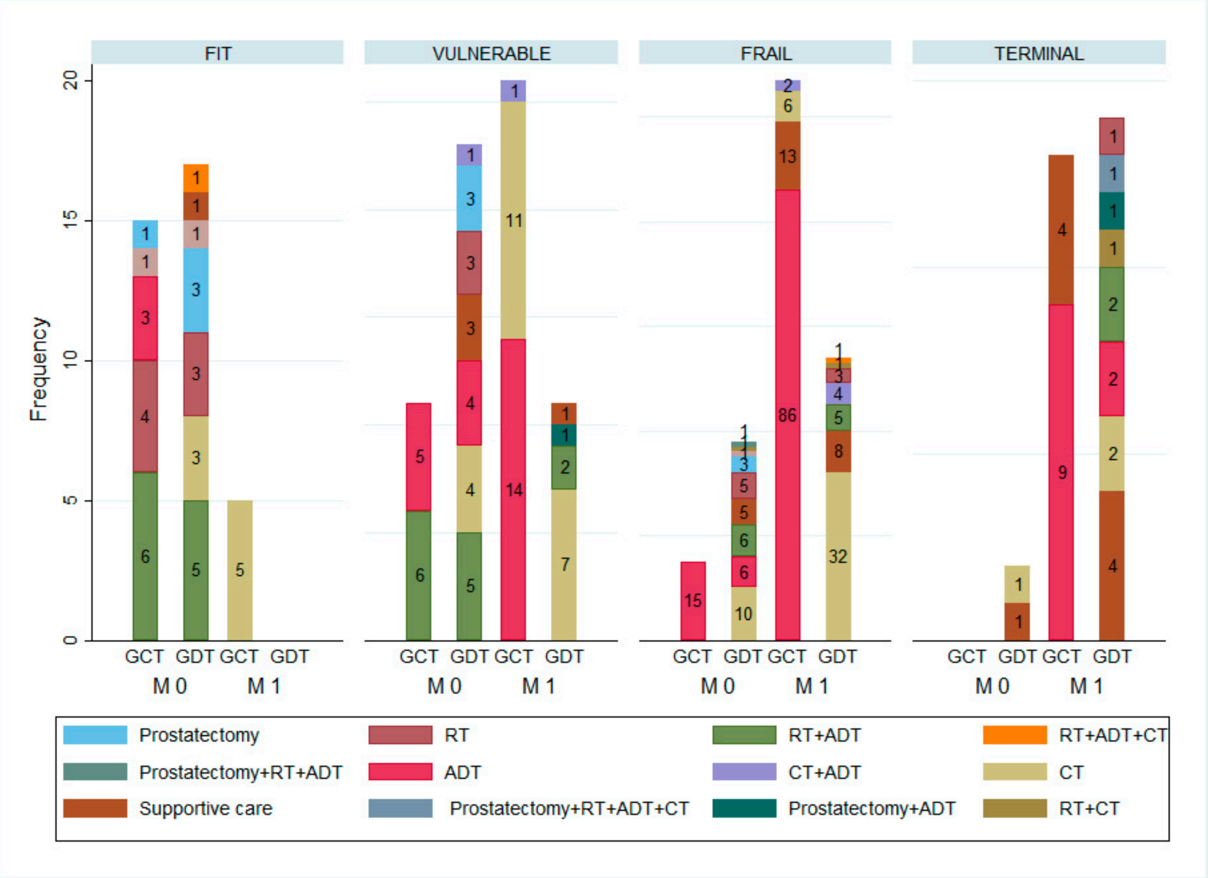

GCT: guideline-concordant treatment, GDT: guideline-discordant treatment, M0: non-metastatic disease, M1: metastatic disease, RT: radiotherapy, ADT: androgen deprivation therapy, CT: chemotherapy.

‡ Fit patients correspond to SIOG 2010 "healthy" patients, SIOG 2014 & SIOG 2017 "fit" patients or Balducci "group 1" patients. Vulnerable patients correspond to SIOG 2010 & SIOG 2014 "vulnerable" patients, SIOG 2017 "frail" patients or Balducci "group 2" patients. Frail patients correspond to SIOG 2010 & SIOG 2014 "frail" patients, SIOG 2017 "disabled" patients or Balducci "group 3" patients. Terminal patients correspond to SIOG 2010 & SIOG 2017 "terminal" patients. SIOG: International Society of Geriatric Oncology.
