# Supplementary material for: Adherence to Treatment Guidelines and Associated Survival in Older Patients with Prostate Cancer: A Prospective Multicentre Cohort Study
Source: Cancers (Basel). 2021 Sep 18;13(18):4694. doi: 10.3390/cancers13184694 (PMC8468518; doi:10.3390/cancers13184694)
Supplement: Supplementary file 1 [file cancers-13-04694-s001.zip › Figure S2.pdf]

Figure S2. Treatments* in the GDT group, stratified by treatment intensity, clinical stage, and health status ‡.

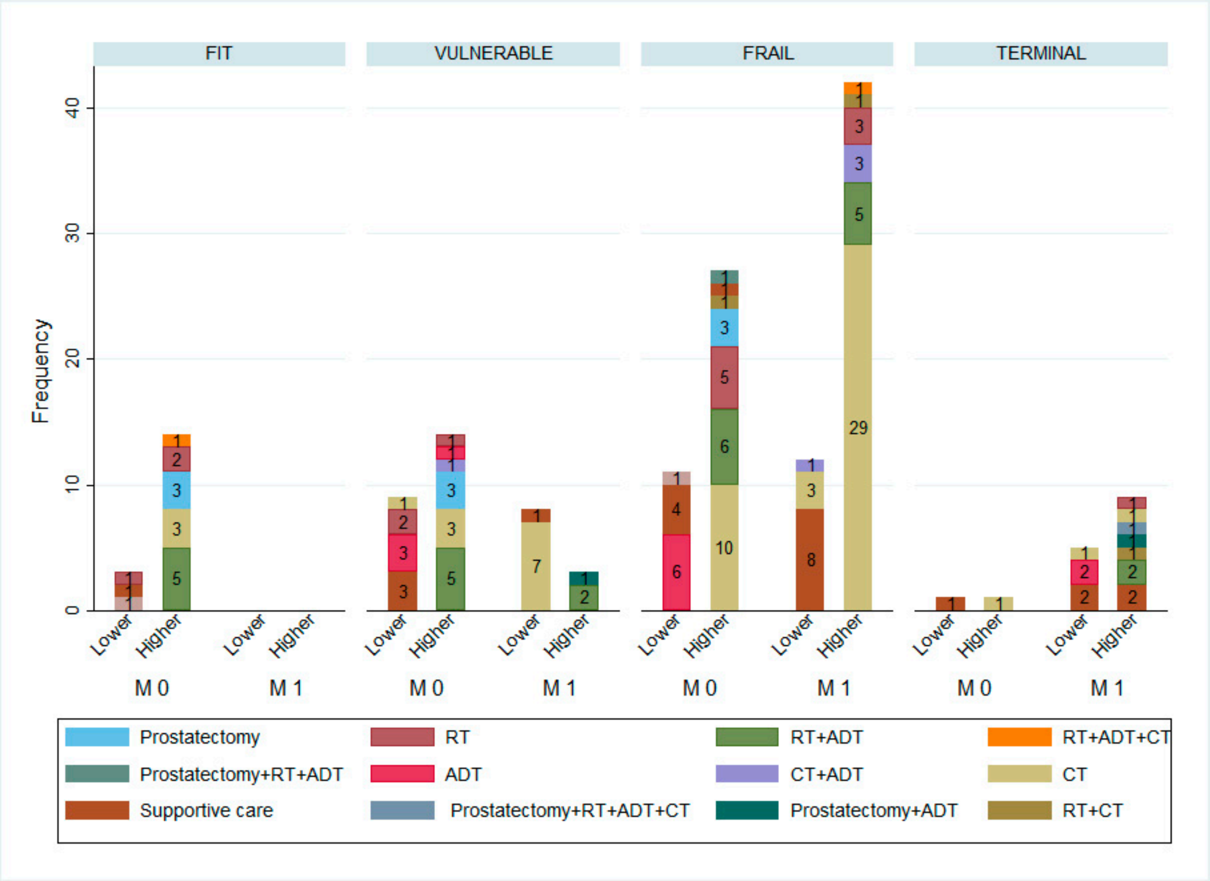

Lower: lower-intensity treatment, Higher: higher-intensity treatment, M0: non-metastatic disease, M1: metastatic disease, RT: radiotherapy, ADT: androgen deprivation therapy, CT: chemotherapy.

***Lower-intensity treatments included non-curative treatments for patients who might receive from intensive cancer treatment or less intense regimens of recommended therapeutic agents (e.g., a fit or Balducci group 1 patient with localized, high-risk prostate cancer, who might receive radiotherapy + ADT but received radiotherapy without ADT). Higher-intensity treatments included treatments of indolent cancers and treatments where toxicity might outweigh the benefit (e.g., a frail or Balducci group 2 patient with localized, intermediate-risk prostate cancer, who might receive active surveillance or watchful waiting but received radiotherapy +ADT).**

‡ Fit patients correspond to SIOG 2010 "healthy" patients, SIOG 2014 & SIOG 2017 "fit" patients or Balducci "group 1" patients. Vulnerable patients correspond to SIOG 2010 & SIOG 2014 "vulnerable" patients, SIOG 2017 "frail" patients or Balducci "group 2" patients. Frail patients correspond to SIOG 2010 & SIOG 2014 "frail" patients, SIOG 2017 "disabled" patients or Balducci "group 3" patients. Terminal patients correspond to SIOG 2010 & SIOG 2017 "terminal" patients. SIOG: International Society of Geriatric Oncology.
