# Supplementary material for: Adherence to Treatment Guidelines and Associated Survival in Older Patients with Prostate Cancer: A Prospective Multicentre Cohort Study
Source: Cancers (Basel). 2021 Sep 18;13(18):4694. doi: 10.3390/cancers13184694 (PMC8468518; doi:10.3390/cancers13184694)
Supplement: Supplementary file 1 [file cancers-13-04694-s001.zip › Figure S3.pdf]

Figure S3. Change over time (2010-2019) in the proportion of GDT in the ELCAPA cohort.

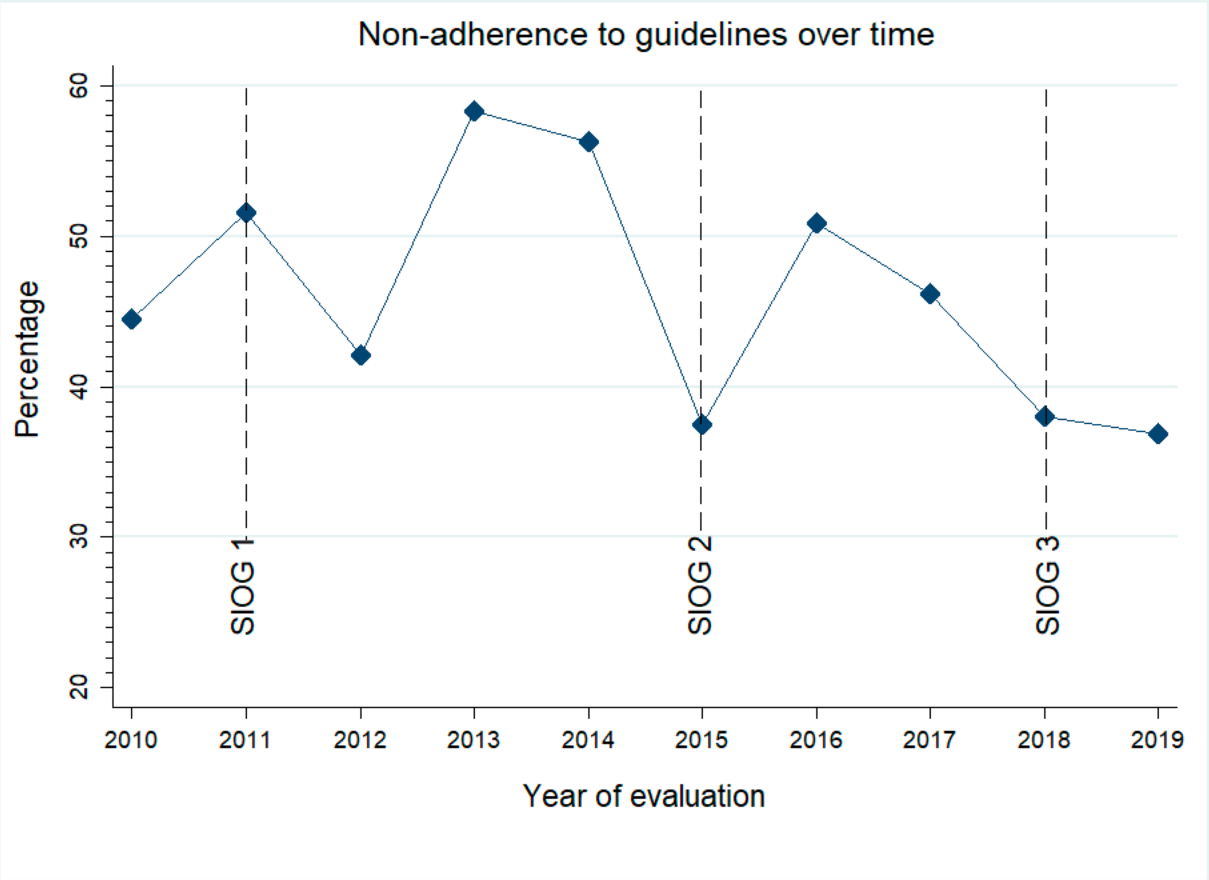

The dashed lines indicate the year after an update in the SIOG's health status classification. SIOG1:2010, SIOG2:2014, SIOG3:2017. Spearman's correlation coefficient was calculated as a guide to the relationship between the proportion of patients with GDT and time (year). There was a statistically significant correlation between GDT and time; overall, the proportion of patients with GDT fell over time (Spearman's correlation coefficient = -0.48, p = <0.001). SIOG: International Society of Geriatric Oncology.
