# Supplementary material for: Adherence to Treatment Guidelines and Associated Survival in Older Patients with Prostate Cancer: A Prospective Multicentre Cohort Study
Source: Cancers (Basel). 2021 Sep 18;13(18):4694. doi: 10.3390/cancers13184694 (PMC8468518; doi:10.3390/cancers13184694)
Supplement: Supplementary file 1 [file cancers-13-04694-s001.zip › Table S1.pdf]

Table S1. Variables used in the guidelines for health status definition, and the classifying algorithm.

Classification[a] (guideline)	Original variables used for health status definition	Variables used for scoring and analysis	Classifying algorithm
**Balducci [1] (adapted) (AFU 2009 [2], AFU 2010 [3], AFU 2013 [4])**	Autonomous patient<br>No comorbidities	≥ 1 abnormal IADL items<br>Number of CIRS-G grade 3 comorbidities.<br>Number of CIRS-G grade 3 comorbidities	Group 1:<br>≥ 1 abnormal IADL items and no CIRS-G grade 3 comorbidities<br>Group 2:<br>≥ 1 abnormal IADL items or ≤2 CIRS-G grade 3 comorbidities and no CIRS-G grade 4 comorbidities<br>Group 3:<br>≥ 1 abnormal IADL items and >2 CIRS-G grade 3 comorbidities or ≥ 1 CIRS-G grade 4 comorbidities<br><br>Treatment decision:<br>Group 1: Standard treatment<br>Group 2: Adjusted treatment<br>Group 3: Supportive care
**Droz et al [5] (SIOG 2010 [5] ; EAU 2012 [6] ; EAU 2013 [7])**	Comorbidities:<br>Any CIRS-G grade 0,1 or 2 comorbidities.<br>1 CIRS-G grade 3 comorbidity.<br>≥ 2 CIRS-G grade 3 comorbidities<br>≥ 1CIRS-G grade 4 comorbidities<br>Dependence:<br>≥ 1 abnormal IADL items (4 items from the Lawton scale: abilities to manage money, manage medications, use transportation, and use the telephone)<br>≥ 1 abnormal ADL items, except for continence (6 items: bathing, dressing, toileting, transferring, continence, and feeding)<br>Nutritional status:<br>Weight loss during the previous 3 months (good nutritional status: <5% of weight loss; risk of malnutrition: weight loss of 5% to 10%; severe malnutrition: weight loss >10%)	The same variables as for the health status definition.<br>Terminal and bedridden (ECOG PS=4)	Healthy:<br>No CIRS-G grade 3 or 4 comorbidities, no abnormal IADL items, no abnormal ADL items, and <5% of weight loss<br>Vulnerable:<br>1 CIRS-G grade 3 comorbidities and no CIRS-G grade 4 comorbidities<br>≥ 1 abnormal IADL items, ≥ 1 abnormal ADL items, and 5 to 10% weight loss<br>Frail:<br>≥ 2 CIRS-G grade 3 comorbidities, ≥ 1CIRS-G grade 4 comorbidities, ≥ 1 abnormal ADL items or >10% weight loss<br>Terminal illness<br>≥ 2 CIRS-G grade 3 comorbidities or ≥ 1CIRS-G grade 4 comorbidities and ≥ 1 abnormal ADL items and >10% weight loss or ECOG PS of 4<br><br>Treatment decision:<br>Healthy: standard treatment<br>Vulnerable: standard treatment except for prostatectomy<br>Frail: adjusted treatment<br>Terminal: supportive care

Table S1. (Continued)

**Droz et al [8] (SIOG 2014 [8]; EAU 2014 [9]; EAU 2015 [10] ; EAU 2016 [11] ; AFU 2016 [12])**	G8 score ≤ 14 of 17<br>Comorbidities:<br>Any CIRS-G grade 0,1 or 2 comorbidities.<br>≥ 1 CIRS-G grade 3 comorbidities<br>≥ 1CIRS-G grade 4 comorbidities<br>Dependence:<br>1 or 2 abnormal ADL items, except for incontinence (6 items: bathing, dressing, toileting, transferring, continence, and feeding)<br>≥ 1 abnormal IADL items (4 items from the Lawton scale: ability to manage money, manage medications, use transportation, and use the telephone)<br>Nutritional status:<br>Weight loss during the previous 3 months (good nutritional status: weight loss <5%; risk of malnutrition: weight loss 5% to 10%; severe malnutrition: weight loss >10%)<br>No neuropsychological problems except for depression.	The same variables as for the health status definition.<br>Cognitive impairment as assessed by an experienced clinician	Fit:<br>G8 score of >14<br>Vulnerable:<br>1 or 2 abnormal ADL items or 5% to 10% weight loss or any CIRS-G grade 0,1 or 2 comorbidities<br>Frail<br>≥ 1 abnormal IADL items or ≥3 abnormal ADL items or >10% weight loss or cognitive impairment or ≥ 1 CIRS-G grade 3 comorbidities or ≥ 1CIRS-G grade 4 comorbidities.<br><br>Treatment decision:<br>Fit: standard treatment<br>Vulnerable: standard treatment<br>Frail: adjusted treatment

Table S1. (Continued)

**Droz et al [13] SIOG 2017 [13]; EAU 2017 [14], EAU 2018 [15])**	G8 score ≤ 14 out of 17<br>Comorbidities:<br>Any CIRS-G grade 0,1 or 2 comorbidities.<br>≥ 1 CIRS-G grade 3 comorbidities<br>≥ 1CIRS-G grade 4 comorbidities<br>Dependence:<br>1 or 2 abnormal ADL items except for incontinence (6 items: bathing, dressing, toileting, transferring, continence, and feeding)<br>Nutritional status:<br>Weight loss during the previous 3 months (good nutritional status <5% of weight loss, risk of malnutrition, weight loss 5% to 10%; severe malnutrition, weight loss >10%)	The same variables as for the health status definition.	Fit:<br>G8 score >14<br>Frail:<br>G8 score ≤ 14 and 1 or 2 abnormal ADL items or 5% to 10% weight loss or any CIRS-G grade 0,1 or 2 comorbidities<br>Disabled:<br>G8 score ≤ 14 and ≥ 3 abnormal ADL items or >10% weight loss or ≥ 1 CIRS-G grade 3 comorbidities or ≥ 1 CIRS-G grade 4 comorbidities.<br>Terminally ill: G8 score ≤ 14 and ≥3 abnormal ADL items and >10% weight loss and ≥ 1 CIRS-G grade 4 comorbidities or ECOG PS of 4.<br><br>Treatment decision:<br>Fit: standard treatment<br>Frail: standard treatment<br>Disabled: adjusted treatment<br>Terminally ill: supportive care

a. For each patient, the health status and the recommended treatment was assessed using the available guidelines in the corresponding period.

We considered that it would have taken a further 12 months before the guidelines were considered for treatment selection. For instance, for a patient included in 2011, the health status and the corresponding treatment was assessed using the AFU guidelines of 2010 and the SIOG guidelines of 2010. The EAU guidelines were not considered for this patient as the EAU guidelines of 2010 did not consider a health status evaluation before treatment selection. For a patient included in 2015, the health status and the corresponding treatment was assessed using the AFU guidelines of 2013, the SIOG guidelines of 2014 and the EAU guidelines of 2014. AFU: French Association of Urology, EAU: European Association of Urology, SIOG: International Society of Geriatric Oncology.

REFERENCES

1. Balducci L, Extermann M. Management of Cancer in the Older Person: A Practical Approach. The Oncologist. 2000;5(3):224-237.
2. Mongiat-Artus P, Peyromaure M, Richaud P, et al. Recommandations pour la prise en charge du cancer de la prostate chez l'homme âgé : un travail du comité de cancérologie de l'association française d'urologie. Prog En Urol. 2009 Dec;19(11):810–7.
3. Salomon L, Azria D, Bastide C, et al. Recommandations en Onco-Urologie 2010 : Cancer de la prostate. Prog En Urol. 2010 Nov;20:S217–51.
4. Salomon L, Bastide C, Beuzeboc P, et al. Recommandations en onco-urologie 2013 du CCAFU : Cancer de la prostate. Prog En Urol. 2013 Nov;23:S69–101.
5. Droz J-P, Balducci L, Bolla M, et al. Management of prostate cancer in older men: recommendations of a working group of the International Society of Geriatric Oncology. BJU Int. 2010 Aug;106(4):462–9.
6. Heidenreich A, Bastian PJ, Bellmunt J, et al. Guidelines on Prostate Cancer 2012 [Internet]. [cited 2020 Nov 9]. Available from: https://uroweb.org/wp-content/uploads/08-Prostate-Cancer_LR-March-13th-20122.pdf
7. Heidenreich A, Bastian, P, Bellmunt J, et al. Guidelines on Prostate Cancer 2013 [Internet]. [cited 2020 Nov 9]. Available from: https://uroweb.org/wp-content/uploads/09_Prostate_Cancer_LR.pdf

8. Droz J-P, Aapro M, Balducci L, et al. Management of prostate cancer in older patients: updated recommendations of a working group of the International Society of Geriatric Oncology. Lancet Oncol. 2014 Aug;15(9):e404-414.
9. Mottet N, Bastian PJ, Bellmunt J, et al. Guidelines on Prostate Cancer 2014 [Internet]. [cited 2020 Nov 9]. Available from: https://uroweb.org/wp-content/uploads/1607-Prostate-Cancer_LRV3.pdf
10. Mottet N, Bellmunt J, Briers E, et al. Guidelines on Prostate Cancer 2015 [Internet]. [cited 2020 Nov 13]. Available from: https://uroweb.org/wp-content/uploads/EAU-Guidelines-Prostate-Cancer-2015-v2.pdf
11. Mottet N, Bellmunt J, Briers E, et al. Guidelines on Prostate Cancer 2016 [Internet]. [cited 2020 Nov 13]. Available from: https://uroweb.org/wp-content/uploads/EAU-Guidelines-Prostate-Cancer-2016-1.pdf
12. Rozet F, Hennequin C, Beauval J-B, et al. Recommandations en onco-urologie 2016-2018 du CCAFU : Cancer de la prostate. Prog En Urol. 2016 Nov;27:S95–143.
13. Droz J-P, Albrand G, Gillessen S, et al. Management of Prostate Cancer in Elderly Patients: Recommendations of a Task Force of the International Society of Geriatric Oncology. Eur Urol. 2017;72(4):521–31.
14. Mottet N, Bellmunt J, Briers E, et al. Guidelines on Prostate Cancer 2017 [Internet]. [cited 2020 Nov 13]. Available from: https://uroweb.org/wp-content/uploads/09-Prostate-Cancer_2017_web.pdf
15. Mottet N, van der Bergh R, Briers E, et al. Guidelines on Prostate Cancer 2018 [Internet]. [cited 2020 Nov 13]. Available from: https://uroweb.org/wp-content/uploads/EAU-ESUR-ESTRO-SIOG-Guidelines-on-Prostate-Cancer-large-text-V2.pdf
