# Supplementary material for: Adherence to Treatment Guidelines and Associated Survival in Older Patients with Prostate Cancer: A Prospective Multicentre Cohort Study
Source: Cancers (Basel). 2021 Sep 18;13(18):4694. doi: 10.3390/cancers13184694 (PMC8468518; doi:10.3390/cancers13184694)
Supplement: Supplementary file 1 [file cancers-13-04694-s001.zip › Table S2.pdf]

Table S2. Demographic characteristics of participants. N=402.

Variable	N (%)
**Decrease in food intake (G8 score item)**	
No decrease	233(58)
Moderate	94(23)
Severe	47(12)
Missing	28(7)
**Weight loss (G8 score item)**	
No weight loss	184(46)
Not known	32(8)
1-3 kg	56(14)
> 3 kg	101(25)
Missing	29(7)
**Mobility (G8 score item)**	
Goes out	265(66)
Able to get out of bed/chair but does not go out	66(16)
Bed- or chair-bound	43 (11)
Missing	28(7)
**Neuropsychological problem (G8 score item)**	
No neuropsychological problem	231(57)
Mild dementia	110(27)
Severe dementia or depression	31(8)
Missing	30(7)
**Body mass index (G8 score item)**	
< 19	22(5)
19-21	32(8)
21-23	60(15)
>23	277(69)
Missing	11(3)
**More than three prescription drugs per day (G8 score item)**	
No	89(22)
Yes	313(78)
**Health status compared with other people of the same age (G8 score item)**	
Better	149(37)
Does not know/As good	173(43)
Not as good	45(11)
Missing	35(9)
**Age (G8 score item)**	
<80	162(40)
80-85	143(36)
> 86	97(24)
**G8 score**	
> 14	89(22)
≤14	303(75)
Missing	10(3)
**Activities of Daily Living score**	
Independent	360 (89.5)
≥ 1 abnormal item	40(10)
Missing	2(0.5)
**Instrumental Activities of Daily Living score**	
Independent	227 (56)
≥ 1 abnormal item	147 (37)
Missing	28(7)

Table S2. (Continued)

**Cumulative Illness Rating Scale for Geriatrics grade 3 or 4 comorbidities**	
No	116(29)
Yes	258(64)
Missing	28(7)
**Eastern Cooperative Oncology Group performance status**	
0-1	213 (53)
2	98 (24.4)
3-4	90(22.4)
Missing	1(0.2)
**Risk of depression**	
No	237 (59)
Yes	135 (34)
Missing	30(7)
**Cognitive impairment**	
No	279 (69)
Yes	115 (29)
Missing	8 (2)
**Health status** [b]	
Fit	54(13)
Vulnerable	81(20)
Frail	230(57)
Terminally ill	30(8)
Missing	7(2)
**Gleason score**	
≤ 6	36 (9)
7	105 (26)
8-10	140(35)
Missing	121(30)
**Serum prostate-specific antigen (ng/ml, median (IQR))**	
30(10.3-122)	325(81)
Missing	77(19)
**Clinical stage**	
Non-metastatic	142 (35)
Metastatic	252 (63)
Missing	8(2)
**Nodal involvement**	
No	190 (47)
Yes	116 (29)
Missing data	96(24)
**D'Amico risk group (if M0, N0 or NX)**	
Low	6 (5)
Intermediate	10(9)
High	69 (59)
Missing	32(27)
**Treatments** [a]	
Radical prostatectomy	17 (4)
Radiotherapy	76 (19)
Androgen deprivation therapy	212 (53)
Chemotherapy	97 (24)
Supportive care	42 (10)

Table S2. (Continued)

**Living alone**	
No	308 (76.7)
Yes	93(23.1)
Missing	1(0.2)
**Marital status**	
Married	281(70)
Other	118(29)
Missing	3(1)
**Smoking status**	
Never smoker	169 (42)
Current smoker	22 (5.5)
Former smoker	157 (39)
Missing	54(13.5)

a. The sum of the percentages may not be 100 because some patients received more than one treatment.

b. Fit patients correspond to SIOG 2010 "healthy", SIOG 2014 & SIOG 2017 "fit" patients or Balducci "group 1" patients. Vulnerable patients correspond to SIOG 2010 & SIOG 2014 "vulnerable" patients, SIOG 2017 "frail" patients or Balducci "group 2" patients. Frail patients correspond to SIOG 2010 & SIOG 2014 "frail" patients, SIOG 2017 "disabled" patients or Balducci "group 3" patients. Terminal patients correspond to SIOG 2010 & SIOG 2017 "terminal" patients. SIOG: International Society of Geriatric Oncology.
