# Supplementary material for: Adherence to Treatment Guidelines and Associated Survival in Older Patients with Prostate Cancer: A Prospective Multicentre Cohort Study
Source: Cancers (Basel). 2021 Sep 18;13(18):4694. doi: 10.3390/cancers13184694 (PMC8468518; doi:10.3390/cancers13184694)
Supplement: Supplementary file 1 [file cancers-13-04694-s001.zip › Table S3.pdf]

Table S3. Bivariate analysis for receiving guideline-discordant treatment, according to at least one of the three sets of guidelines.

	Concordant (%)	Discordant (%)	p-value [a]	OR [95%CI]	p-value [b]
N=356	n= 192	n= 164			
**Decrease in food intake (G8 score item)**					
No decrease	115(63)	87(56)	0.5	1	
Moderate	44(24)	44(29)		1.32 [0.70-2.51]	0.3
Severe	23(13)	23(15)		1.32 [0.80-2.18]	0.4
**Weight loss (G8 score item)**					
No weight loss	92(51)	65(42)	0.3	1	
Does not know	18(10)	12(8)		0.94 [0.43-2.09]	0.9
1-3 kg	26(14)	23(15)		1.25 [0.66-2.39]	0.5
> 3 kg	46(25)	53(35)		1.63 [0.98-2.71]	0.06
**Mobility (G8 score item)**					
Goes out	118(65)	119(77)	0.03	1	
Able to get out of bed/chair but does not go out	40(22)	19(12)		0.47 [0.26-0.86]	0.01
Bed- or chair-bound	24(13)	16(11)		0.66 [0.33-1.31]	0.2
**Neuropsychological problem (G8 score item)**					
No neuropsychological problems	104(58)	102(67)	0.2	1	
Mild dementia	62(34)	39(25)		0.64 [0.39-1.03]	0.1
Severe dementia or depression	15(8)	12(8)		0.82 [0.36-1.83]	0.6
**Body mass index (G8 score item)**					
< 19	13(7)	7(4)	0.7	0.60 [0.23-1.55]	0.3
19-21	15(8)	15(9)		1.11 [0.52-2.37]	0.8
21-23	30(16)	24(15)		0.89 [0.49-1.61]	0.7
>23	129(69)	116(72)		1	
**More than three prescription drugs per day (G8 score item)**					
No	42(22)	38(23)	0.8	1	
Yes	150(78)	126(77)		0.93 [0.56-1.53]	0.8
**Health status compared with other people of the same age (G8 score item)**					
Better	74(42)	57(37)	0.4	1	
Does not know/As good	83(47)	71(47)		1.11 [0.69-1.77]	0.7
Not as good	20(11)	25(16)		1.62 [0.82-3.21]	0.2
**Age (G8 score item)**					
<80	65(34)	78(48)	0.02	1	
80-85	72(37)	55(33)		0.64 [0.39-1.03]	0.1
> 86	55(29)	31(19)		0.47 [0.27-0.81]	0.01
**Activities of Daily Living score**					
Independent	171 (89)	144 (88)	0.8	1	
≥ 1 abnormal item	21 (11)	19 (12)		1.07 [0.56-2.08]	0.8
**Instrumental Activities of Daily Living score**					
Independent	110 (59)	95 (62)	0.7	1	
≥ 1 abnormal item	75 (41)	59 (38)		0.92 [0.59-1.43]	0.7
**Cumulative Illness Rating Scale for Geriatrics grade 3 or 4 comorbidities**					
No	52 (28)	45 (28)	1	1	
At least one	131 (72)	114 (72)		1.01 [0.63-1.61]	1
**Eastern Cooperative Oncology Group performance status**					
0-1	91 (48)	92(56)	0.3	1	
2	52 (27)	37 (23)		0.70 [0.42-1.17]	0.2
3-4	48 (25)	35 (21)		0.72 [0.43-1.22]	0.2
**Risk of depression**					
No	102 (58)	105 (67)	0.1	1	
Yes	75 (42)	52 (33)		0.67 [0.43-1.05]	0.1

Table S3. (Continued)

**Cognitive impairment**					
No	124 (66)	122 (77)	0.02	1	
Yes	65 (34)	37 (23)		0.58 [0.36-0.93]	0.02
**Gleason score**					
≤ 6	12 (10)	17 (14)	0.1	1	
7	40 (32)	49 (42)		0.86 [0.37-2.02]	0.7
8 à 10	73 (58)	51 (44)		0.49 [0.22-1.12]	0.1
**Serum prostate-specific antigen (PSA) level**					
Log PSA	3.8(2.8-4.9)	3.2(2.3-4.8)	0.1	1	
				0.9 [0.79-1.02]	0.1
**Clinical stage**					
Non-metastatic	41 (21)	80 (50)	<0.001	1	
Metastatic	151 (79)	79 (50)		0.27 [0.17-0.43]	<0.001
**Marital status**					
Married	132 (70)	124 (76)	0.2	1	
Other	57 (30)	40 (24)		0.75 [0.47-1.20]	0.2
**Living alone**					
No	149 (78)	130 (79.27)	0.8	1	
Yes	42 (22)	34 (20.73)		0.93 [0.56-1.54]	0.8
**Smoking status**					
Non-smoker	79 (47)	72 (50)	0.8	1	
Active smoker	11 (6)	9 (6)		0.90 [0.35-2.29]	0.8
Ancient smoker	79 (47)	63 (44)		0.88 [0.55-1.39]	0.6

OR: Odds Ratio.

a. Chi squared test for qualitative variables, t-test for quantitative variables.

b. Wald’s test.
