# Supplementary material for: Adherence to Treatment Guidelines and Associated Survival in Older Patients with Prostate Cancer: A Prospective Multicentre Cohort Study
Source: Cancers (Basel). 2021 Sep 18;13(18):4694. doi: 10.3390/cancers13184694 (PMC8468518; doi:10.3390/cancers13184694)
Supplement: Supplementary file 1 [file cancers-13-04694-s001.zip › Table S4.pdf]

Table S4. Bivariate analysis of associated factors with overall survival at 24 and 36 months.

	24-month OS			36-month OS		
	Log rank p	HR [95%CI]	p [a]	Log rank p	HR [95%CI]	p [a]
N= 275						
**G8 score**						
> 14		1			1	
<u><</u>14	<0.001	6.78 [3.45-13.33]	<0.001	<0.001	7.27 [3.82-13.83)	<0.001
**Activities of Daily Living score**						
Independent		1			1	
≥ 1 abnormal item	<0.001	4.48 [2.98-6.74]	<0.001	<0.001	4.66 [3.14-6.92)	<0.001
**Instrumental Activities of Daily Living score**						
Independent		1			1	
≥ 1 abnormal item	<0.001	3.30 [2.36-4.62]	<0.001	<0.001	3.27 [2.37-4.51)	<0.001
**Age**						
<80		1			1	
81-90		1.33 [0.95-1.87]	0.09		1.37 [0.99-1.89)	0.06
>90	<0.001	2.90 [1.69-4.98]	<0.001	<0.001	2.94 [1.72-5.04)	<0.001
**Cumulative Illness Rating Scale for Geriatrics grade 3 or 4 comorbidities**						
No		1			1	
At least one	<0.001	2.38 [1.58-3.58]	<0.001	<0.001	2.62 [1.75-3.90]	<0.001
**Eastern Cooperative Oncology Group performance status**						
0 & 1		1			1	
2		3.29 [2.19-4.96]	<0.001		3.41 [2.31-5.01]	<0.001
3&4	<0.001	6.69 [4.52-9.91]	<0.001	<0.001	6.54 [4.48-9.55]	<0.001
**Risk of depression**						
No		1			1	
Yes	<0.001	1.95 [1.40-2.70]	<0.001	<0.001	1.94 [1.41-2.67]	<0.001
**Cognitive impairment**						
No		1			1	
Yes	<0.001	2.09 [1.50-2.92]	<0.001	<0.001	2.13 [1.55-2.93]	<0.001
**Weight loss**						
<5%		1			1	
5-10%		1.51 [0.98-2.32]	0.06		1.60 [1.05-2.42]	0.03
>10%	<0.001	2.73 [1.86-3.99]	<0.001	<0.001	3.03 [2.10-4.34]	<0.001
**Gleason Score**						
<u><</u> 6		1			1	
7		1.52 [0.63-3.70]	0.3		1.24 [0.56-2.71]	0.6
8 to 10	<0.001	3.24 [1.39-7.52]	0.01	<0.001	2.70 [1.29-5.66]	0.01
**Serum prostate-specific antigen (PSA) level**						
Log PSA	<0.001*	1.34 [1.20-1.48]	<0.001	<0.001 [b]	1.32 [1.19-1.46]	<0.001
**Clinical stage**						
Non-metastatic		1			1	
Metastatic	<0.001	4.81 [3.14-7.36]	<0.001	<0.001	4.88 [3.27-7.30]	<0.001
**Smoking status**						
Non-smoker		1			1	
Active smoker		1.03 [0.49-2.14]	>0.9		0.93 [0.45-1.93]	0.8
Ancient smoker	0.6	1.19 [0.84-1.68]	0.3	0.6	1.17 [0.84-1.63]	0.4

Table S4. (Continued)

**Treatment according to all 3 guidelines**						
Concordant		1			1	
Discordant	0.8	0.97 [0.71-1.34]	0.8	0.8	0.96 [0.70-1.30]	0.8
**Treatment according to the SIOG guidelines**						
Concordant		1			1	
Discordant	0.8	1.06 [0.73-1.54]	0.8	0.8	0.95 [0.66-1.38]	0.8
**Treatment according to the EAU guidelines**						
Concordant		1			1	
Discordant	0.9	0.99 [0.66-1.48]	0.9	0.9	0.96 [0.66-1.42]	0.9
**Treatment according to the AFU guidelines**						
Concordant		1			1	
Discordant	0.8	1.00 [0.72-1.39]	1	0.8	0.96 [0.70-1.32]	0.8

HR: hazard ratio.

a. Wald's test.

b. p-value corresponds to the Cox regression model.
